# Cooperative effect of silicon and other alloying elements on creep resistance of titanium alloys: insight from first-principles calculations

**DOI:** 10.1038/srep30611

**Published:** 2016-07-28

**Authors:** Yang Li, Yue Chen, Jian-Rong Liu, Qing-Miao Hu, Rui Yang

**Affiliations:** 1 Institute of Metal Research, Chinese Academy of Sciences, 72 Wenhua Road, Shenyang 110016, China; 2 Department of Mechanical Engineering, The University of Hong Kong, Pokfulam Road, Hong Kong SAR

## Abstract

Creep resistance is one of the key properties of titanium (Ti) alloys for high temperature applications such as in aero engines and gas turbines. It has been widely recognized that moderate addition of Si, especially when added together with some other elements (X), e.g., Mo, significantly improves the creep resistance of Ti alloys. To provide some fundamental understandings on such a cooperative effect, the interactions between Si and X in both hexagonal close-packed α and body-centered cubic β phases are systematically investigated by using a first-principles method. We show that the transition metal (TM) atoms with the number of d electrons (*N*_*d*_) from 3 to 7 are attractive to Si in α phase whereas those with *N*_*d*_ > 8 and simple metal (SM) alloying atoms are repulsive to Si. All the alloying atoms repel Si in the β phase except for the ones with fewer d electrons than Ti. The electronic structure origin underlying the Si-X interaction is discussed based on the calculated electronic density of states and Bader charge. Our calculations suggest that the beneficial X-Si cooperative effect on the creep resistance is attributable to the strong X-Si attraction.

Due to their high specific strength, good damage tolerance, and excellent corrosion resistance, titanium (Ti) alloys are excellent candidates for application in aero engines and gas turbine components to improve the thrust weight ratio. In such applications, Ti alloys suffer from high temperature and sustained stress, with which, the slow plastic deformation (i.e., creep) develops gradually. The development of the creep deformation reduces the distance between the blades and the crankcase of the engine such that it lowers the working efficiency. Furthermore, the accumulation of the creep deformation may finally result in creep fracture, which shortens the serving life of the engine. Therefore, improving the creep resistance is one of the key issues for the design of high temperature Ti alloys.

It has been widely recognized that the addition of Si improves significantly the creep resistance of Ti alloys. Most high temperature Ti alloys contain Si^1^,^2^,^3^, and there usually exists an optimal content of Si. Excess addition of Si reduces the creep resistance or plasticity and ductility of the alloy. The optimal contents for Ti6242, Ti-6Al-3Sn-3Zr, and BT9 alloys were reported to be about 0.09%, 0.30%, and 0.28%, respectively^4^,^5^,^6^. To rationalize the observed experimental phenomena, different mechanisms have been proposed in the literature: (1) Solution strengthening. Dislocation attracts strongly the Si atoms, leading to the formation of Cottrell atmosphere, which suppresses the climb of the dislocation^7^,^8^; (2) Precipitation strengthening. With Si content over the solubility limit, precipitated phases such as Ti_5_Si_3_ and Ti_2_Si form during aging and impede the slip of the dislocations and grain boundaries^9^,^10^,^11^. Although moderate amount of silicide precipitates improve the creep resistance of the alloys, this effect is very limited since the precipitates may distribute heterogeneously on the grain/phase boundaries of the alloys, which destroy the compatibility of the plastic deformations among different crystal grains such that decreasing the plasticity and ductility of the alloys (i.e., the thermal stability)^1^,^7^,^12^,^13^. Therefore, in order to obtain the optimal creep resistance strengthening effect, a higher solid solubility of Si is preferred and the silicide precipitates should be as fine and dispersive as possible.

Experiments have demonstrated that Si improves more efficiently the creep resistance of the Ti alloys when it is added together with some other elements such as Mo and W^14^. Nonetheless, the underlying physics is still unrevealed. According to the mechanisms of the Si-strengthening effects as described above, it is reasonable to conceive that the cooperative effects of Si and the other alloying elements on the creep resistance may be ascribed to: (a) The alloying atoms serve as the heterogeneous nuclei for the silicide precipitates and help to form fine and dispersed precipitate phases; (b) The alloying atoms trap the Si atoms, which hampers the long range diffusion of Si, suppresses the formation of silicide precipitates, and increases the solid solubility of Si; (c) The Si and alloying atoms form clusters, interacting strongly with the dislocations such that the dislocation climb is further inhibited. In order to clarify the above hypothesises, understanding of the interaction between Si and the alloying atom is crucial.

In order to study the cooperative effects of Si and other alloying elements on the creep resistance of Ti alloys, in the present work, the interaction energy between Si and the other alloying atom is systematically investigated by using a first-principles method. The alloying elements cover all of the transition metal (TM) elements in the 4th, 5th, and 6th periods of the Periodic Table (from group 3 to group 12), as well as the simple metal (SM) elements from the 3rd to the 6th period. Both hexagonal close-packed (hcp) *α* phase and body centered cubic (bcc) *β* phase are considered.

## Results

### Distance dependence of interaction energy

The interaction energy between Si and the alloying atom X is defined as^15^,^16^


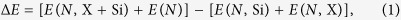


where *E*(*N,* X + Si), *E*(*N,* Si), and *E*(*N,* X) are the total energies of the *N*-site supercells containing a Si-X pair, a Si atom, and an X atom, respectively. *E*(*N*) is the total energy of the N-site supercell of pure Ti. [*E*(*N,* X + Si) + *E*(*N*)] represents the energy of a 2N site system with Si and X atoms interacting with each other whereas [*E*(*N,* Si) + *E*(*N,* X)] represents the energy of the 2N site system with non-interacting Si and X. Therefore, the energy difference between the two systems measures the X-Si interaction. A negative value of ∆*E* implies that the X and Si atoms are attractive to each other.

**Figure 1:**
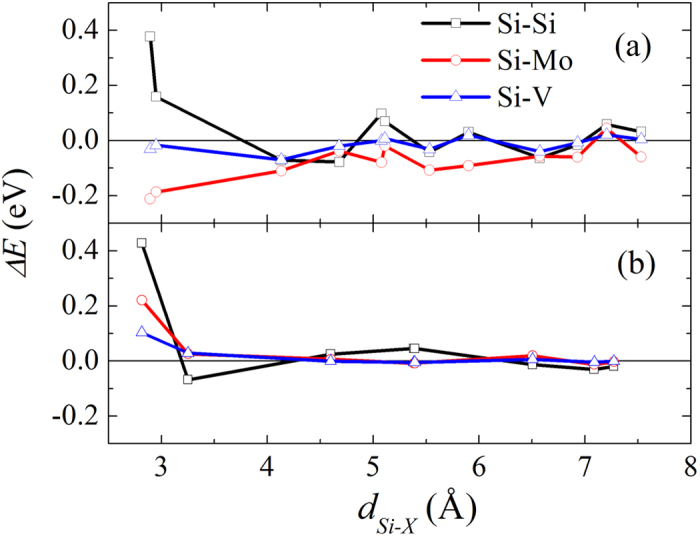
The interaction energy ∆*E* between Si and alloying atoms X (X = Si, Mo and V) against the Si-X distance (*d*_Si−X_) in both α-(a) and β-Ti (b).

The interaction energy ∆*E* depends on the distance between Si and the alloying atom X. The distance dependence of the interaction energy ∆*E* is checked taking Si-Si, Mo-Si, and V-Si as the examples. As seen in Fig. 1, the interaction energy ∆*E* converges to nearly zero with increasing X-Si distance and the strongest interaction occurs at the nearest neighbor distance except for the V-Si pair. The convergence of ∆*E* for the α phase is much slower than that for the β phase. For the α phase, ∆*E* still has a sizable value of about ±0.10 eV at the 4th neighbor distance for Si-Si and Mo-Si, and the interaction remains evident up to the 8th neighbor distance. For the β phase, the interaction energy ∆*E* is close to zero beyond the 2nd neighbor distance. In other words, the X-Si interaction is longer ranged in the α phase than in the β phase. The reason could be that the structure of the α phase (hcp) is close-packed whereas the structure of the β phase (bcc) is relatively more open.

In both α and β phases, the Si-Si interaction energy is positive at the nearest neighbor (NN) distance and negative at the next nearest neighbor (NNN) distance. Thus, Si atoms are repulsive to each other at the NN distance and attractive at NNN distance, which, according to Friedel theory^17^, explains the fact that ordered silicides form easily in Ti alloys. The alternating interaction energies between positive and negative values for the Si-Si pair do not occur for the Mo-Si and V-Si pairs. In α-Ti, Si and Mo atoms are essentially attractive to each other as shown by the negative interaction energies. The V-Si interaction in the α phase is very weak for all distances. In β-Ti, both Mo and V are repulsive to Si at the nearest neighbor distance.

### X-Si pair interaction

Since the strongest X-Si interaction usually appears at the NN distance, we focus ourselves the NN X-Si pair interaction in this section.

**Figure 2:**
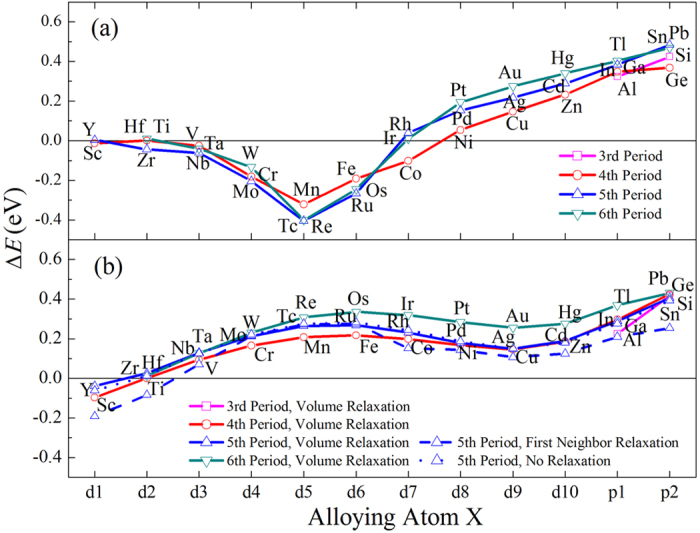
The interaction energy between Si and alloying atom X (∆*E*) in α-Ti (a) and β-Ti (b).

In the α phase, as seen in Fig. 2a, for the transition metal (TM) alloying elements in the same row of the periodic table, the interaction energy ∆*E* is nearly zero for the early elements with number of d electrons *N*_*d*_ of 1 and 2. ∆*E* decreases with increasing *N*_*d*_, reaches a minimum at *N*_*d*_ = 5, and increase with further increasing *N*_*d*_. For *N*_*d*_ in between 3 and 7, Δ*E* is negative. For the simple metal alloying (SM) elements, Δ*E* is positive and increases with the number of p electrons *N*_*p*_. Negative ∆*E* indicates that X and Si are attractive to each other while positive value means they are repulsive. The larger the absolute value of ∆*E*, the stronger the interactions. Therefore, the Si and TM alloying atoms with *N*_*d*_ in between 3 and 7 are attractive to each other, among which the strongest attraction occurs for Si-X_*d*5_ (namely X = Mn, Tc and Re) pair.

The bcc β-Ti, as the high temperature phase, transforms to other artificial structures when it is fully relaxed during the first-principles calculation. Therefore, we have performed relaxation with different constraint conditions. Figure 2b shows the interaction energy ∆*E* of X-Si in β-Ti. The solid and the dotted lines represent the calculations with and without volume relaxations, respectively. The dash line represents the relaxations of the NN sites of both Si and X. The interaction energy ∆*E* calculated with different constraints for the elements in the 5th period shows similar behaviors. Particularly, the calculations with and without volume relaxations generate almost identical ∆*E*. When the NN sites of Si and X are relaxed, ∆*E* becomes smaller. This is obvious because the relaxation lowers the energy of the supercell. Moreover, the radius of X in the late 5th period is much smaller than Ti, the relaxation is thus decrease more effectively the repulsion between Si and X.

The X-Si interaction energy ∆*E* in β phase shows a wave-like behavior with increasing *N*_*d*_ and *N*_*p*_. The X-Si interaction energies ∆*E* are positive for all the alloying elements except for those with *N*_*d*_ = 1 and 2. Namely, the atoms with N_*d*_ > 2 are repulsive to Si in β-Ti.

### X-2Si cluster interaction

**Figure 3:**
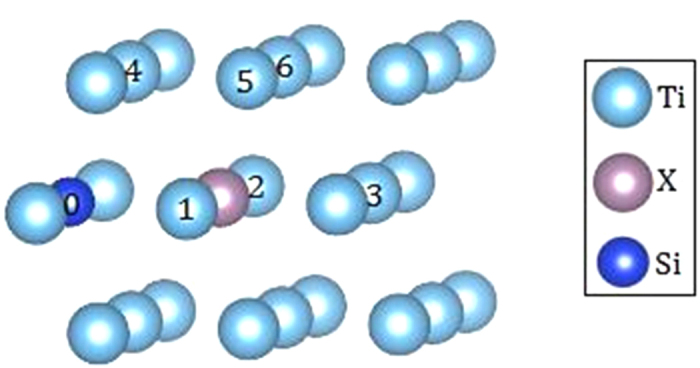
The configurations of X-2Si clusters in α-Ti. The numbered sites from 0 to 3 are in the middle plane whereas 4, 5 and 6 are in the upper plane.

In order to further understand whether alloying atom X can attract more Si atoms so as to promote the formation of precipitates in α-Ti, we calculate the interaction energy between X-Si_I_ pair and another Si atom (Si_II_). Here, we take X = Mo and Nb as representative examples. The configurations of X + 2Si clusters are shown in Fig. 3. Both Si_I_ and Si_II_ are the NNs of X whereas the distance between Si_I_ and Si_II_ varies.

We first consider the situation that X-Si_I_ traps an isolated Si_II_ in α-Ti, where the interaction energy is defined as


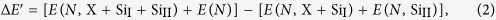


with *E*(*N*, X + Si_I_ + Si_II_) being the total energy of the *N*-site supercell containing X + Si_I_ + Si_II_ cluster. This situation corresponds to the case that the Si concentration is relatively high and there exist some excess isolated Si atoms which are not trapped by X. The interaction Δ*E*′ for different X + 2Si configurations are listed in Table 1.

It is seen from Table 1, for both Mo and Nb, when the two Si atoms are the NN (configurations 0–1 and 0–4 with distances of 2.95 and 2.89 Å, respectively) and 4^th^ NN (configurations 0–2 and 0–6 with distance of 5.08 and 5.11 Å, respectively) of each other, we get repulsive interaction between X-Si_I_ and Si_II_ with positive interaction energy Δ*E*′. The repulsion is obviously due to the repulsive interaction between the Si atoms. The strongest repulsions with Δ*E*′ of 0.377 eV for Mo and 0.255 eV for Nb occur for the NN Si_I_ and Si_II_ with distance of 2.89 Å, both weaker than the Si-Si repulsive energies (Δ*E* about 0.400 eV) in Ti-2Si. This may be ascribed to the attraction of Mo/Nb to Si_II_.

When the two Si atoms are the 2^nd^ NN (with distance of 4.13 Å, configuration 0–5) and 6^th^ NN (with distance of 5.90 Å, configuration 0–3) of each other, the interaction energy Δ*E*′ between Ti-Si_I_ and Si_II_ is negative, namely, X-Si_I_ and Si_II_ are attractive to each other. For the 2^nd^ NN Si_I_ and Si_II_ atoms, the attraction between Mo-Si_I_ and Si_II_ with Δ*E*′ of −0.182 eV in the Ti-Mo-2Si system are stronger than that between two Si atoms in the Ti-2Si system (Δ*E* of about −0.100 eV). This is understandable since Mo and Si_I_ are both attractive to Si_II_ in this case. The interaction between Nb-Si_I_ and Si_II_ is almost at the same level as that between two Si atoms in the Ti-2Si system because the Nb-Si interaction is relatively weak.

Second, we consider the situation that X-Si_I_ traps Si_II_ in another X-Si_II_ pair, where the interaction energy is defined as


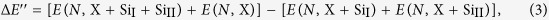


This situation corresponds to the case that the Si concentration is relatively low and all Si atoms are assumed to already form X-Si pairs in α-Ti. Therefore, an X-Si pair has to be broken in order to form an X-2Si cluster.

The interaction energy Δ*E*′′ is also listed in Table 1. It is seen that, for Mo, Δ*E*′′ is positive for all the configurations, indicating that the Mo-Si pairs are energetically more favorable than the Nb-2Si cluster. The interaction energies Δ*E*′′ of all the configurations of Mo-2Si are larger than those of Mo-2Nb. The reason is that the Mo-Si pair interaction is stronger than the Nb-Si pair so that it is more difficult to break the Mo-Si pair than the Nb-Si pair. For Nb-2Si, Δ*E*′′ of the configuration with Si_I_ and Si_II_ 2^nd^ nearest to each other (0–5) is negative, implying that a Nb-Si pair may take a Si atom in another Nb-Si pair to form Nb-2Si cluster.

From the above calculations, we may conclude that the alloying atoms X which are attractive to Si may trap several Si atoms to form clusters at relatively high Si concentration. However, the number of the trapped Si atoms is limited by the repulsive Si-Si interaction. At low Si concentration, the strong Si-trapping alloying atoms such as Mo prefer to form pairs with Si whereas the weaker Si trapping alloying atoms such as Nb may still attract multiple Si atoms to form clusters. Such a difference between the strong and weak Si-trapping alloying atoms may help to explain the different effects of these alloying elements on the creep resistance of Si containing titanium alloys as discussed later in this paper.

## Discussion

### Electronic structure origin

In the previous sections, we see that the interaction energy ∆*E* of X-Si changes regularly with the number of valence electrons. In order to explore the underlying mechanisms, the electronic density of states (DOS) and Bader charges of some representative systems are calculated.

**Figure 4:**
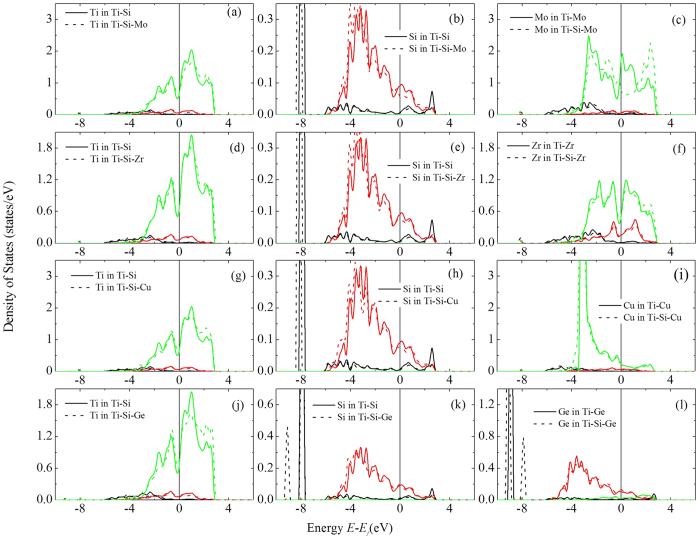
Partial density of states of Ti (**a,d,g,j**), Si (**b,e,h,k**), and X (**c,f,i,l**) in α-Ti-Si/X and α-Ti-Si-X systems (X = Mo, Zr, Cu, Ge). The vertical lines in the figures represent the Fermi level.

Figure 4(a,b,c) presents the partial DOS of the atoms in Ti-Si/Mo and Ti-Si-Mo systems, where Si and Mo attract quite strongly with each other. It is seen that the DOSs of Ti (Fig. 4a) atoms in Ti-Si and Ti-Mo-Si systems show little difference. Actually, the DOS (especially the d DOS) of Ti in Ti-Si is very similar to that in pure α-Ti (not shown). A new s state of Si in α-Ti (as compared to the pure Si, not shown) shows up at about −8.0 eV (Fig. 4b). The new s sates should not form through the hybridization between the states of Si and Ti since it is absent in the DOS of Ti. We consider that the formation of the new s state of Si is ascribed to the donation of electrons from the surrounding Ti atoms. This is confirmed by our Bader charge analysis which demonstrates that Si gains about 1.66 electrons from the Ti atoms. Each of the NN Ti atoms of Si donates about 0.10 electrons. Therefore, Coulomb electrostatic attraction contributes to the bonding between Ti and Si such that the Ti-Si bond shows some ionic character. Another contribution is from the metallic interaction as some states present at Fermi level for both Si and Ti.

There exists a peak right at the Fermi level for the p DOS of Si in Ti-Si (Fig. 4b) and the d DOS of Mo in Ti-Mo (Fig. 4c). When Mo-Si pair forms in α-Ti, this peak disappears and a pseudogap is opened at the Fermi level for both Si and Mo due to the hybridization between Si-p and Mo-d states, indicating the covalent interaction between Mo and Si atoms. On the other hand, the height of the low-lying s states of Si is not affected by the addition of Mo, and Si atom gains 1.71 electrons, slightly more than that in binary Ti-Si alloy (See Table 2). Mo also gains 1.66 electrons from its surrounding Ti atoms. Therefore, Columb electrostatic repulsion should present between Si and Mo. The Mo-Si attraction might be ascribed to the stronger Mo-Si covalent bond than the Ti-Si bond such that Si prefers to stay nearest to Mo instead of Ti, and the Mo-Si electrostatic repulsion could not override the attraction.

Figure 4(d,e,f) presents the partial DOS of the atoms in Ti-Si/Zr and Ti-Zr-Si systems where Si and Zr atoms show almost no observable interaction. Again, the DOS of Ti remains almost unchanged in Ti-Si and Ti-Zr-Si systems (Fig. 4d). The sp states of Si are shifted slightly to lower energy due to the addition of Zr (Fig. 4e) whereas the DOS of Zr in Ti-Zr and Ti-Zr-Si shows little difference (Fig. 4f). The Bader charge analysis shows that Zr atom remains electronic neutral such that the Zr-Si ionic attraction should be weaker than Ti-Si one, which raises the energy of Ti-Zr-Si system, indicating Zr-Si repulsive interaction. However, the energy raising is canceled somehow by the increasing Ti-Si ionic attraction since Si gains 0.13 more electrons from its surrounding Ti atoms in Ti-Zr-Si than that in Ti-Si. This explains the very weak interaction between Zr and Si in α-Ti.

Figure 4(g,h,i) presents the partial DOS of the atoms in Ti-Si/Cu and Ti-Cu-Si systems where Si and noble metal Cu atoms are repulsive to each other. The main features of the DOSs of Ti and Si are similar to those in Fig. 4(d,e,f) for Ti-Zr-Si system. The DOS of Cu in Ti-Cu is not significantly affected by the addition of Si. It is noted that the d DOS of Cu forms a very high and narrow peak below Fermi level, indicating that the d electrons of Cu are mainly localized. Therefore, these electrons contribute little to the metallic bonding between Cu and other atoms. Namely the Cu-Si metallic bond should be weaker than the Ti-Si one such that the site nearest to Ti instead of Cu is favorable to Si and we get repulsive interaction between Cu and Si. Both Si and Cu atoms gain electrons from Ti atoms. Thus, the electrostatic repulsion occurs between Si and Cu. This further contributes to the Cu-Si repulsive energy.

The simple metal alloying atoms such as Ge are also repulsive to Si in α-Ti. However, the underlying physics is somehow different from that for the noble metal atom Cu. As shown in Fig. 4(l), when isolated in α-Ti, Ge shows a low-lying s peak due to the donation of electrons from Ti to Ge, similar to the case of Ti-Si system. Ge gains about 1.57 electrons from the surrounding Ti atoms. Consequently, Si and Ge are repulsive to each other because of the Coulomb electrostatic repulsion when Si and Ge atoms are next to each other. Furthermore, the low-lying s peak splits when Si and Ge atoms exist as a NN pair. This means that the Si-s and Ge-s states hybridize to form a bonding state and an anti-bonding state. Both the bonding state and anti-bonding state are below Fermi level, and, therefore, are occupied by electrons. This hybridization costs energy which may also contribute to the repulsion between Si and Ge atoms.

**Figure 5:**
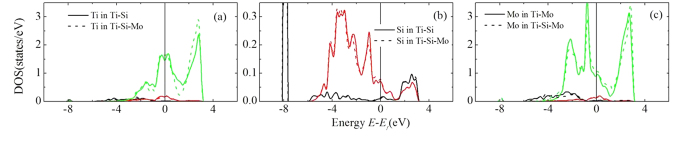
Partial density of states of Ti (**a**), Si (**b**), and Mo in β-Ti-Si/Mo and β-Ti-Mo-Si systems. The vertical lines in the figures represent the Fermi level.

As discussed previously in this section, the attractive interaction between Si and the alloying atoms X with *N*_*d*_ from 2 to 7 is mainly ascribed to the strong X-Si covalent bonding. In β-Ti, the covalent bonding is absent. As shown in Fig. 5 where the DOS of the Ti-Mo-Si system is presented as a representative example, there is no Si-p−Mo-d hybridization induced pesudogap at the Fermi level, dissimilar to the case of the α phase. Therefore, the X-Si interaction is dominated by the metallic and ionic interactions. The DOS of Mo at Fermi level is lower than that of Ti such that the Mo-Si metallic bond is weaker than the Ti-Si one, which results in repulsive Mo-Si interaction in β-Ti. On the other hand, both Mo and Si gain electrons from Ti (see Table 2). Subsequently, there exists electrostatic repulsion between Mo and Si. Namely, both metallic and ionic effects contribute to the repulsion between Mo and Si. The same mechanism applies to the interactions between Si and other transition metal atoms. The electronic structure origin between Si and other simple atoms in β-Ti is the same as that for the α-phase. We do not present the DOSs for other systems for the sake of conciseness of the paper.

### X-Si interaction and creep resistance

The titanium alloys developed for high temperature applications are mainly “near α” alloys (such as IMI834 and Ti-6242, with varying Si content) containing 10–15% β phase. Experiments have demonstrated that the creep of this kind of titanium alloys is controlled by the deformation in the α phase^18^. Therefore, the interactions between Si and other alloying atoms in α phase influence directly the creep resistance of these high temperature titanium alloys.

As we mentioned in the introduction of this paper, the creep resistance strengthening effect originates largely from Si in solid solution state. From our calculations, the alloying atoms with 3 ≤ N_*d*_ ≤ 7, e.g., Nb, Mo, Tc, Ta, W and Re are attractive to Si in α-Ti. Consequently, these alloying elements may trap Si atoms and limit the long-range diffusion of Si atoms, which increases the solid solubility of Si in α-Ti, and, therefore, strengthens the creep resistance. The alloying atoms attracting more strongly the Si atoms are expected to increase the creep resistance more efficiently because of two facts. First, stronger X-Si attraction limits the long-range diffusion of Si atoms more effectively. Second, our calculations of the interaction energies of X-2Si clusters demonstrate that the X-Si pairs are favorable to the X-2Si cluster at low Si concentration for the strong X-Si attraction. This indicates that dislocation may get more pins in case of the strong X-Si attraction than that for the weak one, assuming that X distributes homogenously in the matrix. Such effect may account for the cooperative effect of Mo/W and Si on the creep resistance of titanium alloys. Our calculations predict that Tc and Re attract Si most strongly among all the alloying elements. Therefore, their creep resistance hardening effects are expected to be the best when adding together with Si in high temperature titanium alloys. Another advantage of the strong attractors of Si is that they are beneficial to the thermal stability of titanium alloys because the formation of silicides may be impressed.

It should be noted that some alloying atoms such as Cr, Mn, Fe and Co may also trap Si. However, experiments have demonstrated that these alloying elements are harmful to the creep resistance of titanium alloys at high temperature^19^,^20^,^21^. The reason is that these alloying atoms are fast diffusers in titanium and enhance the self-diffusion of Ti atoms. Therefore, they accelerate the dislocation climb of titanium alloys during the creep.

The elements with *N*_*d*_ from 8 to 10 have repulsive interactions with Si in α phase, so they cannot trap Si, and, therefore, cannot improve the creep resistance of high temperature Ti alloys. These elements are seldom added to Ti alloys in experiments.

High temperature titanium alloys generally contain about 4∼8% Al and 2∼5% Sn^2^. These simple metal alloying elements also enhances the creep resistance of titanium alloys although they are repulsive to Si. The beneficial effect of Al and Sn is irrelevant to Si. Similar to Si, simple metal alloying atoms such Al are expected to form short-range ordered structure or Al-rich precipitates in α-Ti since they repel to each other at the NN distance but attract at 2^nd^ NN distance. According to experiments, the addition of Sn may avoid the formation of long-range ordered brittle α_2_ phase, and, therefore, improves the thermal stability of high temperature Ti alloys^22^,^23^.

Although the creep resistance is mainly determined by the dislocation movement in α phase, the repulsion between Si and the transition metal alloying atoms, especially the β stabilizers such as V, Nb, Mo, W, etc., may influence the creep resistance indirectly. The β stabilizers are mainly distributed in the β phase. The repulsion between Si and the β stabilizers moves the Si atoms out of the β phase such that the Si atoms accumulate at the α/β phase boundaries, which may increase the creep resistance of the alloy.

## Conclusions

Using a first-principles plane-wave pseudopotential method, we calculated the interaction energy between Si and other alloying atoms in both in α and β titanium. We showed that, in α-Ti, the interactions between Si and the transition metal (TM) alloying atoms with the number of d electrons *N*_*d*_ of 1 or 2 are very weak. The TM alloying atoms with from 3 to 7 are attractive to Si, and the strongest attraction occurs for *N*_*d*_ = 5 (namely, Mn, Tc, and Re). The TM alloying atoms with *N*_*d*_ > 8 and simple metal (SM) alloying atoms are repulsive to Si. In β-Ti, almost all alloying atoms are repulsive to Si except the ones with *N*_*d*_ = 1 and 2. Further calculations of the interaction energy between the X-Si pair and Si demonstrated that, for those TM atoms attractive strongly to Si (e.g. Mo), Si exist as X-Si pairs instead of X-2Si clusters at relatively low Si content whereas for those TM atoms weakly attractive to Si (e.g. Nb), the X-2Si clusters is more stable.

The calculated electronic density states and Bader charges demonstrated that the attraction between Si and the TM alloying atoms (X) in α-Ti is mainly ascribed to the strong covalent bonding due to the hybridization between Si-p and X-d states, whereas the repulsion between Si and other TM alloying atoms in both α and β titanium may be ascribed to the weaker X-Si metallic bond than the Ti-Si one and the X-Si electrostatic repulsion. The repulsion between Si and the SM alloying atoms is due to the unfavorable hybridization between their low-lying s states and the electrostatic repulsion.

Based on the calculated interaction energies between the alloying atoms and Si, we proposed that the beneficial cooperative effect of Si and some alloying atoms on the creep resistance of titanium alloys is attributable to the strong attraction of the alloying atoms to Si which increases the solid solubility of Si in titanium alloys.

## Methods

The total energies are calculated by using a plane-wave pseudpotential method based on density functional theory (DFT), implemented in Vienna Ab initio Simulation Package (VASP). The projector augmented waves (PAW) potential is adopted to describe the electron-core interaction. The plane-wave cutoff energy is set as 500 eV. The energy tolerance of the electronic minimization is 1 × 10^–6^ eV. A 4 × 4 × 3 supercell of the conventional unit cell is constructed, and the *k*-point mesh is set as 3×3×3 for both α and β phase. For the hcp *α* phase, the geometry of the supercells including lattice parameters and atomic positions are fully-optimized with the Hellman-Feynman force tolerance of 0.01 eV/Å. Since the *β* phase is a high temperature phase and is not stable at T = 0 K, the atoms deviate significantly from the original lattice sites after a full relaxation. Two different levels of optimizations have been performed. The first level is relaxing only the cell volume with atomic positions and cell shape fixed; the second level is the relaxation of the nearest neighbor sites of Si and X with the cell volume and shape fixed.

## Additional Information

**How to cite this article**: Li, Y. *et al*. Cooperative effect of silicon and other alloying elements on creep resistance of titanium alloys: insight from first-principles calculations. *Sci. Rep.* **6**, 30611; doi: 10.1038/srep30611 (2016).

## Figures and Tables

**Table 1:** The interaction energies Δ*E*′ and Δ*E*′′ between X + Si_I_ pair and Si_II_ for different X + Si_I_  + Si_II_ configurations in α-Ti.

Configuration	*d*_SiI-SiII_ (Å)	ΔE′		ΔE′′	
		X = Mo	X = Nb	X = Mo	X = Nb
0–1	2.95	0.163	0.085	0.380	0.099
0–2	5.11	0.149	0.102	0.366	0.117
0–3	5.90	−0.016	−0.011	0.201	0.003
0–4	2.89	0.377	0.255	0.575	0.299
0–5	4.13	−0.182	−0.081	0.016	−0.037
0–6	5.08	0.089	0.031	0.287	0.075

**Table 2:** Electron transfer (in e) of the Ti-X-Si system from Bader charge analysis.

System	Si	X
α-Ti-Si	+1.66	/
α-Ti-Si-Mo	+1.71	+1.66
α-Ti-Si-Zr	+1.79	−0.00
α-Ti-Si-Cu	+1.67	+1.14
α-Ti-Si-Ge	+1.57	+1.71
β-Ti-Si	+1.70	/
β-Ti-Si-Mo	+1.58	+1.53
β-Ti-Si-Zr	+1.70	+0.10
β-Ti-Si-Cu	+1.15	+1.57
β-Ti-Si-Ge	+1.50	+1.50

## References

[b1] Winstone M. R., Rawlings R. D. & West D. R. F. The creep behaviour of some silicon-containing titanium alloys. Journal of the Less Common Metals 39, 205–217 (1975).

[b2] Es-Souni M. Creep behaviour and creep microstructures of a high-temperature titanium alloy Ti–5.8 Al–4.0 Sn–3.5 Zr–0.7 Nb–0.35 Si–0.06 C (Timetal 834): part I. Primary and steady-state creep. Materials characterization 46, 365–379 (2001).

[b3] Jia W., Zeng W., Zhou Y., Liu J. & Wang Q. High-temperature deformation behavior of Ti60 titanium alloy. Materials Science and Engineering: A 528, 4068–4074 (2011).

[b4] Lefranc P., Doquet V., Gerland M. & Sarrazin-Baudoux C. Nucleation of cracks from shear-induced cavities in an α/β titanium alloy in fatigue, room-temperature creep and dwell-fatigue. Acta Materialia 56, 4450–4457 (2008).

[b5] Wang S., Liu J. & Chen D. Tensile and fatigue properties of electron beam welded dissimilar joints between Ti–6Al–4V and BT9 titanium alloys. Materials Science and Engineering: A 584, 47–56 (2013).

[b6] Rivière J., Pichon L., Drouet M., Poquillon D. & Galdikas A. Silicon based coatings deposited by dynamic ion mixing for oxidation protection of a Ti6242 alloy. Surface and Coatings Technology 201, 8343–8347 (2007).

[b7] Paton N. E. & Mahoney M. Creep of titanium-silicon alloys. Metallurgical Transactions A 7, 1685–1694 (1976).

[b8] Kehoe M. & Broomfield R. The mechanisms by which certain solute elements improve the creep strength of alpha titanium. Titanium science and technology, 2167–2178 (1973).

[b9] Mitra R. Microstructure and mechanical behavior of reaction hot-pressed titanium silicide and titanium silicide-based alloys and composites. Metallurgical and Materials Transactions a-Physical Metallurgy and Materials Science 29, 1629–1641 (1998).

[b10] Poletaev D. O. . Ab initio-based prediction and TEM study of suicide precipitation in titanium. Computational Materials Science 95, 456–463 (2014).

[b11] Colinet C. & Tedenac J.-C. Structural stability of intermetallic phases in the Si-Ti system. Point defects and chemical potentials in D8(8)-Si3Ti5 phase. Intermetallics 18, 1444–1454 (2010).

[b12] Venkatramani G., Ghosh S. & Mills M. A size-dependent crystal plasticity finite-element model for creep and load shedding in polycrystalline titanium alloys. Acta Materialia 55, 3971–3986 (2007).

[b13] Eylon D., Fujishiro S., Postans P. J. & Froes F. High-temperature titanium alloys—a review. JOM 36, 55–62 (1984).

[b14] Chao L. . Shenyang Hengyunda Titanium Ind Dev Co.

[b15] Hu Q. M., Xu D. S. & Li D. First-principles investigations of the solute-vacancy interaction energy and its effect on the creep properties of alpha-titanium. Philos. Mag. A-Phys. Condens. Matter Struct. Defect Mech. Prop. 81, 2809–2821 (2001).

[b16] Hu Q. M., Xu D. S., Yang R., Li D. & Wu W. T. First-principles investigations of ordering in binary α-Ti solid solutions. Philosophical Magazine 83, 217–229 (2003).

[b17] Tütto I. & Zawadowski A. Quantum theory of local perturbation of the charge-density wave by an impurity: Friedel oscillations. Physical Review B 32, 2449 (1985). 10.1103/physrevb.32.2449 9937320

[b18] Viswanathan G., Karthikeyan S., Hayes R. & Mills M. Creep behaviour of Ti-6Al-2Sn-4Zr-2Mo: II. Mechanisms of deformation. Acta materialia 50, 4965–4980 (2002).

[b19] Herzig C., Mishin Y. & Divinski S. Bulk and interface boundary diffusion in group IV hexagonal close-packed metals and alloys. Metallurgical and Materials Transactions A 33, 765–775 (2002).

[b20] Mishra H., Satyanarayana D., Nandy T. & Sagar P. Effect of trace impurities on the creep behavior of a near α titanium alloy. Scripta Materialia 59, 591–594 (2008).

[b21] Gale W. F. & Totemeier T. C. Smithells metals reference book. (Butterworth-Heinemann, 2003).

[b22] Ramachandra C. & Singh V. Precipitation of the ordered Ti_3_Al phase in alloy Ti-6.3Al-2Zr-3.3 Mo-0.3Si. Scripta metallurgica 20, 509–512 (1986).

[b23] Rosenberg H. (Titanium Metals Corp. of America, Henderson, Nev., 1970).

